# Evaluation of the performance of existing non-laboratory based cardiovascular risk assessment algorithms

**DOI:** 10.1186/1471-2261-13-123

**Published:** 2013-12-28

**Authors:** Jacob K Kariuki, Eileen M Stuart-Shor, Suzanne G Leveille, Laura L Hayman

**Affiliations:** 1 College of Nursing and Health Sciences, University of Massachusetts, Boston, USA; 2 Beth Israel Deaconess Medical Center, Boston, Massachusetts, USA

**Keywords:** Global risk assessment, Risk assessment algorithms, Discrimination, Calibration, Absolute cardiovascular risk

## Abstract

**Background:**

The high burden and rising incidence of cardiovascular disease (CVD) in resource constrained countries necessitates implementation of robust and pragmatic primary and secondary prevention strategies. Many current CVD management guidelines recommend absolute cardiovascular (CV) risk assessment as a clinically sound guide to preventive and treatment strategies. Development of non-laboratory based cardiovascular risk assessment algorithms enable absolute risk assessment in resource constrained countries.

The objective of this review is to evaluate the performance of existing non-laboratory based CV risk assessment algorithms using the benchmarks for clinically useful CV risk assessment algorithms outlined by Cooney and colleagues.

**Methods:**

A literature search to identify non-laboratory based risk prediction algorithms was performed in MEDLINE, CINAHL, Ovid Premier Nursing Journals Plus, and PubMed databases. The identified algorithms were evaluated using the benchmarks for clinically useful cardiovascular risk assessment algorithms outlined by Cooney and colleagues.

**Results:**

Five non-laboratory based CV risk assessment algorithms were identified. The Gaziano and Framingham algorithms met the criteria for appropriateness of statistical methods used to derive the algorithms and endpoints. The Swedish Consultation, Framingham and Gaziano algorithms demonstrated good discrimination in derivation datasets. Only the Gaziano algorithm was externally validated where it had optimal discrimination. The Gaziano and WHO algorithms had chart formats which made them simple and user friendly for clinical application.

**Conclusion:**

Both the Gaziano and Framingham non-laboratory based algorithms met most of the criteria outlined by Cooney and colleagues. External validation of the algorithms in diverse samples is needed to ascertain their performance and applicability to different populations and to enhance clinicians’ confidence in them.

## Background

Cardiovascular disease (CVD) continues to be the leading cause of morbidity and mortality in the developed world despite abundance of resources and well developed health care systems [1]. In recent years, a new trend has been observed in developing countries where cardiovascular disease has become the overall leading cause of death due in part to the ongoing epidemiological transition from infectious to non-communicable diseases. Currently 80% of the global burden of CVD is in developing countries [2-4].

This epidemiological transition has introduced a protracted double burden of disease in developing countries which are also plagued by underdeveloped and fragile health care systems [2]. In the backdrop of the looming public health crisis, most governments in developing countries such as Sub-Saharan Africa still allocate 80% of their total health budgets to acute communicable diseases [5]. The same trend of skewed allocation of resources has been followed by major donor agencies including the World Health Organization (WHO). A comparative analysis of WHO 2008–2009 budget by Stuckler and collegues [5] revealed that only 12% of WHO total budget was earmarked for non-communicable diseases, while 87% was allocated for infectious diseases.

The high burden and increasing prevalence of CVD in resource constrained countries necessitates that robust and pragmatic primary and secondary prevention strategies be implemented with urgency. Many current CVD management guidelines recommend absolute cardiovascular (CV) risk assessment as a clinically sound guide to preventive and treatment strategies [3,6]. Calculating the patient’s absolute risk for CVD enables clinicians to estimate the likelihood that a particular constellation of risk factors will contribute to the development of a CVD related morbidity or mortality over a specific period of time [7,8].

Absolute risk estimates can be useful in raising CVD awareness, and in motivating adherence to recommended lifestyle changes or treatment. In clinical practices across many developed countries, CV risk assessment algorithms have been mainly used to identify individuals at high risk for developing CVD within a specified period of time, usually 10 years, and to select those individuals for more intensive preventive and treatment interventions [8].

A proactive preventive strategy that incorporates the absolute risk approach could potentially have a major impact in lowering the incidence and the burden of CVD in resource constrained countries. By directing the scarce resources toward those in greatest need, the disease burden associated with CVD could be reduced without the expense of unnecessary treatment and associated adverse effects to those at low risk [3]. However, while the absolute risk approach could be beneficial in resource constrained countries, the widely used CV risk assessment algorithms are based on laboratory measures that are not readily available in resource constrained countries [9,10].

In recent years, there have been significant efforts to develop non-laboratory based cardiovascular risk assessment algorithms that are feasible for resource constrained countries. However, unlike their laboratory based counterparts, the non-laboratory based algorithms have not been critically evaluated using rigorous criteria. The purpose of this systematic review of the literature is to evaluate the performance of existing non-laboratory based CV risk assessment algorithms using the benchmarks for clinically useful CV risk assessment algorithms outlined by Cooney and colleagues [6].

## Methods

A literature search was performed in MEDLINE, CINAHL, Ovid Premier Nursing Journals Plus, and PubMed databases. The key words used in the search were: Non-laboratory based CVD risk assessment algorithms OR Non-laboratory based CVD risk function OR Non-laboratory based CVD risk assessment system OR simple office-based CVD risk prediction function. Additionally, a search for related articles was done within the databases and using the bibliographies of the selected articles. The inclusion criteria required the full text report to be written in English, focused on algorithms for primary prevention of CVD, and have human subjects aged nineteen years old and above. All the included articles were based on studies which had ethical clearance from the relevant institutional review boards. We excluded secondary sources and studies focusing on algorithms for secondary prevention of CVD and special populations. No other limits were employed. The search strategy was guided by the PRISMA model [11].

The methodological soundness of each of the indentified CVD risk assessment algorithms was evaluated using the benchmarks for clinically useful cardiovascular risk assessment algorithms outlined by Cooney and colleagues [6] shown in Table 1.

**Table 1:** Cooney’s criteria for evaluating clinically useful risk assessment algorithms

1	Appropriateness of statistical methods used to derive the function.
	• Representativeness of the algorithm’s derivation sample, optimal statistical power and methods, and clarity of end point predicted by the function.
2	Performance of the function: internal and external validity.
	• Discrimination, calibration, and sensitivity of the algorithm(s) in the derivation and external datasets.
3	Usability of the algorithm.
	• Impact of an algorithm’s format on its use and uptake in clinical settings.
4	Inclusion of appropriate risk factors.
	• Inclusion of major risk factors known to be prevalent in the target population.
5	Measurable health gains associated with the use of the algorithm(s).
	• Tangible clinical benefits associated with use of the algorithm(s).

The appropriateness of the statistical methods used to derive the function was measured by analyzing the representativeness of the algorithm’s derivation sample, statistical power, statistical methods used, and end point predicted by the function. Performance was evaluated by analyzing discrimination (using Area under Receiver Operating Characteristics), calibration (using Hosmer-Lemeshow goodness of fit testing), and sensitivity of the algorithms in their derivation and external datasets. Usability was assessed by weighing the impact of algorithm’s format on its use in clinical settings. Inclusion of appropriate risk factors was evaluated by assessing the incorporation in the algorithm of major risk factors which are known to be prevalent in the target population. Measurable health gains that have been associated with the use of the algorithms were measured by assessing any tangible clinical benefits associated with use of the algorithms.

## Results

**Figure 1:**
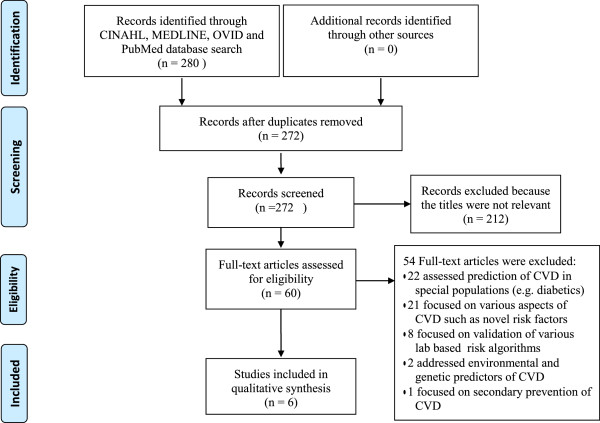
**PRISMA flow diagram.** Outlines the literature search flow.

When the search criteria were applied, MEDLINE yielded 3 articles, CINAHL 1 article, and PubMed 6 articles. All the articles retrieved in MEDLINE and CINAHL were relevant, while two of those retrieved from PubMed were excluded because they were editorial reviews. When the same search criteria were applied in Ovid Premier Nursing Journals Plus, 270 articles were retrieved. Of the total 280 articles, 274 (97.9%) could not be included in the primary review because they failed to meet the inclusion/exclusion criteria after reviewing their titles and abstracts. The PRISMA diagram in Figure 1 describes the flow of the search. The six relevant articles identified focused on five non-laboratory based CV risk assessment algorithms namely: Framingham non-laboratory based algorithm [12], Gaziano non-laboratory based algorithm [9,13], WHO/ISH non-laboratory based algorithms [14], Swedish Consultation-based method [15], and the UK General Practice model [16].

The five identified non-laboratory based cardiovascular risk assessment algorithms were developed for use in primary prevention of CVD in resource constrained settings [9,12,14-16]. Table 2 outlines the covariates, end points and risk categories of the five non-laboratory based CV risk assessment algorithms. The identified five algorithms were critically appraised using Cooney’s criteria for evaluating clinically useful risk assessment algorithms [6] outlined in Table 1 and described under the methods section.

**Table 2:** Covariates, end points and risk categories of non-laboratory based CV risk prediction algorithms

**Algorithms**	**Covariates**								**Endpoints**	**Risk categories**
	**Sex**	**Age**	**Smoking**	**BP**	**HTN treatment**	**BMI or W/H ratio**	**Diabetes**	**Health history**		
Non-laboratory based-Framingham [12]	M or F	30-74	• Yes, current smoker	Systolic 120-160	• Yes to current treatment	kg/m^2^	• Yes, on insulin or oral hypoglycemic medications, or FBS ≥126 mg/dl	NA	10-year risk of general and individual CVD events (coronary, cerebro-vascular, and peripheral arterial disease and heart failure).	0-6%,
										6-20%,
										>20%
			• No, never/former smoker		• No current treatment		• No, none of the above criteria			
Non-laboratory based-Gaziano [9]	M or F	35-74	• Yes, current/former smoker	Systolic 111- 180	• Yes to current treatment	kg/m^2^	• Yes, diabetes self reported	NA	5-year risk for first-time fatal and non-fatal cardiovascular disease events.	≪5%
										5–10%
										>10–20%
										>20–30%
			• No, never		• No current treatment		• No, diabetes not self reported			>30%
Non-laboratory based-WHO/ISH [14]	M or F	40-70	• Yes, current/ former smoker ≪1 yr	Systolic 140-180	NA	NA	• Yes, on insulin or oral hypoglycemic drugs; or FBS ≥126 mg/dl; or postprandial plasma glucose 200 mg/l on two occasions.		10-year combined risk for acute myocardial infarction and stroke (Fatal and nonfatal).	≪10%,
										10-?≪?20%
										20-?≪?30%
										30-?≪?40%
										≥40%
			• No, never/former smoker >1 yr				• No, none of above criteria.			
Swedish consultation based method [15]	M or F	40-59	• Yes, current.	Systolic ≥140 or Diastolic ≥90	• Yes to current treatment	waist/height ratio	• Yes, diabetes self reported	Family hx of CVD (angina, MI and stroke)	Time to first fatal or nonfatal CVD, which include; cardiovascular death, angina, MI, CABG, PTCA, stroke and PAD.	Not given
					• No current treatment		• No, diabetes not self reported			
UK general practice model [16]	F	60-79	• Current	Systolic 123-173	NA	NA	NA	Self-rated health	CHD and CVD events which include MI, CABG or angioplasty and stroke.	Not explicit
			• Former							
			• Never							

### Evaluating the algorithms using Cooney’s criteria

#### Appropriateness of methods used to derive the function

According to Cooney and colleagues [6] a clinically useful CV risk assessment algorithm should be derived from an adequately powered sample which is representative of the population to which the algorithm is to be applied. In addition, valid statistical methods should be employed, and the end point(s) predicted by the algorithm should be clearly defined to enable standardization across populations. Table 3 outlines the sample characteristics, statistical methods and validation of the non-laboratory based CV risk assessment algorithms.

**Table 3:** Sample characteristics, statistical methods and validation of non-laboratory based CV risk prediction algorithms

**Algorithms**	**Methods**	**Internal validation**			**External validation**
Framingham non-lab based algorithm [12]	*Design*: Prospective cohort study of Framingham heart study and Framingham offspring study.		Men	Women	No external validation reported
		Discrimination (C-statistics):	0.749	0.785	
		Calibration (χ^2^)	13.61	10.24	
	*Sample*: 8491 participants (4522 women) aged 30 to 74 years who were free of CVD.	Sensitivity/specificity (20%, 10 yrs risk threshold)	(48/85)%	(58/83)%	
	*Baseline data*: 1968 to 1971, 1971 to 1975, 1984 to 1987	Comparative analysis [12] (General CVD risk)			
			Non-lab Framingham vs. Lab-Framingham-D’Agostino		
		C-statistics (*men*)	0.749	0.763	
		C-statistics (*women*)	0.785	0.793	
		Calibration χ^2^ (men)	13.61	13.48	
	*Analysis*: Cox proportional-hazards regression	Calibration χ^2^ (women)	10.24	7.79	
Gaziano non-lab based algorithm [9]	*Design*: Prospective cohort study of NHANES I Epidemiologic Follow-up Study (NHEFS)		Men	Women	*C-statistics:* 0.782 Men 0.807 Women
					*Calibration* (χ2): Not assessed
		C-statistics:	0.783	0.831	
		Calibration: (χ^2^)	6.61	3.45	
	*Sample*: 6186 subjects (3349 Women) aged between 25 to 74 yrs without CVD or cancer.	Sensitivity/specificity:			*Comparative analysis*: Gaziano algorithm was compared with 4 lab based algorithms as follows: Framingham-D’Agostino-2008; Framingham-Anderson; [20] SCORE low; SCORE high [21].
		30%, 5 yrs risk threshold:	(8.8/98.6)%	(5.1/99.5)%	
		20%, 5 yrs risk threshold:	(24.8/93.7)%	(17.6/97.7)%	
	*Baseline data*: 1971 and 1975,1982–84,1987, and 1992	Comparative analysis [9] (CVD risk)			*C-statistics* (men) respectively:
			Gaziano vs. Lab-Framingham-Anderson [20]		0.782; 0.772; 0.778; 0.785; and 0.784.
	*Analysis*: Cox proportional-hazards regression	*C-statistic* (men)	0·821	0.820	*C-statistics* (women) respectively:
					0.807; 0.832; 0.821; 0.792; and 0.793.
		*C-statistic* (women)	0·860	0.858	
		χ (men*)*	6.61	6.70	
		χ^2^ (women)	3.45	6.62	
WHO/ISH cardiovascular risk prediction charts [14]	*Design*: Relative risks associated with CV risk factors were obtained from the comparative risk assessment project; these were combined with estimated absolute risks for each WHO sub region based on global burden of disease study.	C-statistics: Not reported.			No external validation reported
	*Sample*: from theoretical dataset.	Calibration (χ2*):* Not reported.			
	*Baseline data*: Not specified				
	*Analysis*: Not specified.				
Swedish consultation based method [15].	*Design*: A cross-sectional, population-based screening study with 17-year follow-up in Southern Sweden.	C-statistics (Overall):		0.794	
		Calibration (χ^2^):		Not reported.	
	*Sample*: 689 individuals (349 men) without CVD.	Sensitivity/specificity.		Not reported	
	*Baseline data*: 1989 to May 1990	Comparative analysis [15]			No external validation reported
			Consultation vs. SCORE [21]		
	*Analysis*: Cox proportional-hazards regression	*C-statistic*:	0.794	0.767	
		Calibration (χ^2^)	Not reported.		
		Consultation vs. extensive lab method [15].			
		*C-statistic:*	0.794	0.806	
		Calibration (χ^2^):	Not reported.		
UK General Practice (GP) model [16].	*Design*: Prospective cohort study of British Women Heart and Health study.		CHD	CVD	No external validation reported
		C-statistics*:*	0.66	0.67	
		Calibration (χ^2^)	Not reported		
	*Sample*: 3582 women aged 60 to 79 years without CVD.	Sensitivity/specificity:			
		30%, 10 yrs risk threshold:	(10/95)%	(38/79)%	
	*Baseline data*: 1999 to 2001	15%, 10 yrs risk threshold:	(44/74)%	(85/30)%	
	*Analysis*: Weibull proportional hazards survival model.	Comparative analysis [16]			
			GP model vs. Framingham [20]		
		*C-statistic:*	0.67	0.66	
		Calibration (χ^2^)	Not reported.		
			GP model vs. expanded Framingham [16].		
		*C-statistic:*	0.66	0.64	
		Calibration (χ^2^)	Not reported.		

##### 

**Sample characteristics of the algorithms** The *Gaziano non-laboratory based algorithm* was derived from a sample of 6186 participants of the first National Health and Nutrition Examination Survey Epidemiologic Follow-up Study (NHEFS) who were free from CVD and cancer [9]. NHEFS was a prospective cohort study of NHANES I participants aged between 25 to 74 years at their initial assessment between 1971 and 1975 [17]. The specific sample used to derive the Gaziano non-laboratory based algorithm included 3349 women and 2837 men who were ethnically and racially diverse [9]. Therefore, this algorithm may be applied across ethnic and racial groups aged 25 to 74 years.

*The WHO/ISH non-laboratory based algorithm*s have no specified sample. Rather they were formulated using a hypothetical dataset created for each of the six WHO regions based on the risk factor prevalence mapped by an earlier Collaborative Risk Assessment Project [3]. It is therefore difficult to ascertain whether the hypothetical dataset reflected characteristics typical of the populations targeted by the WHO/ISH non-laboratory based algorithms, and whether the algorithm would perform as projected.

The *Swedish Consultation based method* was derived from a sample of 689 individuals, from Southern Sweden, without CVD at baseline. The population-based approach of the study ensured that all inhabitants aged 40 to 59 years in So¨dera°kra, Southern Sweden were invited to participate in the cardiovascular risk-factor screening project. The sample was balanced according to gender, with 340 women and 349 men participating [15]. However, since the algorithm was derived in a relatively middle aged white population, its applicability to older and racially diverse populations is unknown. In addition, the sample may not be adequately powered since it is relatively small compared to the samples used to derive other algorithms.

The *UK General practice model* was derived from a prospective cohort sample of 3582 participants of the British Women Heart and Health study [16]. All the participants in this study were women aged 60 to 79 years without CVD at baseline. As such, the UK General practice model may not be well suited to predict CV risk in younger women and male populations, but it is likely to be more precise with the older female populations compared to the other four algorithms.

The *Framingham non-laboratory based algorithm* was derived from a population based sample of 8491 Framingham study participants and their offspring who were free from CVD [12]. A total of 4522 women and 3969 men aged 30 to 74 years were included in the study. At the inception of the study, the town of Framingham was an industrial trading center inhabited by white middle class families [18]. Consequently, the Framingham study participants are considered representative of the white middle class population in the North Eastern United States.

Since the Framingham, UK General Practice model and Swedish Consultation based method were derived from predominantly white samples, questions remain unanswered about their applicability in racially/ethnically diverse population. Taken at face value, these algorithms may be construed to be inappropriate for non-white populations. In their systematic review of risk scoring methods, Beswick and colleagues [10] reported that Framingham-based algorithms had poor calibration in certain ethnic groups, and underestimated risk in socio-economically deprived populations. Whereas the applicability of Framingham based algorithms to different populations continues to be debated, proponents of broad applicability algorithms to diverse populations cite the INTERHEART study’s revelation that the nine major modifiable risk factors for CVD account for up to 90% risk of MI incident across a wide range of cultural, ethnical and geographical regions around the globe [1,4].

The idea of broad applicability of algorithms to diverse populations has been supported by Beswick and colleagues [10]. Although they reported poor performance of Framingham based algorithms in certain ethnicities, such as the Hispanics, they also observed that the algorithms performed reasonably well in predicting coronary heart disease death and myocardial infarction for white and black populations within 5 years of follow-up. In addition, recalibration of Framingham functions enhanced predicted levels of absolute risk in these populations. Therefore, there is convincing evidence that CV risk prediction algorithms can be applicable in populations with different racial and ethnic profiles from their derivation samples.

##### 

**Statistical methods and endpoints** The Swedish Consultation based method, the non-laboratory based Framingham and Gaziano algorithms were derived using Cox proportional-hazards regression [9,12,15] while the UK General Practice model was derived using Weibull proportional hazards survival model [16]. The WHO/ISH non-laboratory based algorithms have not specified the statistical methods used to derive their functions. Overall, Cox and Weibull proportional-hazards regression approaches are considered superior to logistic regression because they account for variable follow-up times and losses to follow-up. However, unlike the Weibull method which imposes a parametric function on the baseline survival, the Cox method has an additional advantage of not making assumptions regarding the shape of the underlying survival [6,19]. Therefore, the latter approach may be more suitable for this application.

The endpoints of the Framingham non-laboratory based algorithm are clearly described as general and individual fatal and non-fatal CVD events that include; coronary, cerebrovascular and peripheral arterial disease, and heart failure [12]. On the other hand, the Gaziano non-laboratory based algorithm endpoints are first time fatal and non-fatal CVD events that include myocardial infarction, stroke, congestive heart failure, and coronary revascularization [9]. The WHO/ISH non-laboratory based algorithms endpoints are specified as fatal and non-fatal acute myocardial infarction and stroke [3], while the Swedish Consultation based method endpoints are first fatal or nonfatal CVD, which include; cardiovascular death, angina, myocardial infarction, coronary artery bypass graft surgery, percutaneous transluminal angioplasty, stroke and peripheral arterial disease [15]. The UK General Practice model endpoints are coronary heart disease and CVD events which include myocardial infarction, coronary artery bypass graft surgery or angioplasty and stroke [16].

The endpoints for the Framingham non-laboratory based algorithm were ascertained through hospitalization records, physical examinations, medical records, and communication with personal physicians; [12] while the endpoints for the Gaziano non-laboratory based algorithm were established through review of mortality records, hospitalization records, medical history, medical records, pathology reports, and electrocardiographs [9]. The Swedish Consultation model endpoints were ascertained through review of cardiovascular mortality and in-hospital care for CVD; [15]while the UK General Practice model endpoints were confirmed through a follow-up medical record review [16]. Table 2 outlines endpoints of the five non-laboratory based CV risk assessment algorithms.

Whereas all the five non-laboratory based algorithms have clearly defined their endpoints, significant distinctions exist. The Gaziano non-laboratory based algorithm, GP model and the WHO/ISH non-laboratory based algorithms use a combination of CHD/CVD events as their endpoints, while the Framingham non-laboratory based algorithm and the Consultation model focus on general CVD risk rather than hard coronary events. Cooney and colleagues criteria [6] considers general CVD risk as the most appropriate primary endpoint since atherosclerosis may be manifest outside the coronary vessels, for instance as stroke or peripheral vascular disease. Another significant characteristic of the Framingham non-laboratory based algorithm is that it retains the ability to estimate the risk of cause-specific outcomes such as cerebrovascular events. This is important because cause-specific outcomes such as stroke are important in low-risk countries and older populations [19].

#### Performance of the algorithms

Performance of a cardiovascular risk assessment algorithm is assessed through internal and external validation. The main approaches for measuring the performance include discrimination, calibration, and reclassification [6].

Discrimination is the ability of an algorithm to assign a higher risk to those who will develop the end point compared to those who will not, and it is frequently measured using Area Under Receiver Operating Characteristic Curve (AUROC) or Harrell’s C statistic. An AUROC of 1 denotes perfect discrimination whereas 0.5 equates to chance. Although the C statistic of CV risk assessment algorithms rarely exceeds 0.8, a commendable algorithm should have a C statistic of 0.75 or higher [6,16]. In addition threshold discrimination, operationalized by sensitivity and specificity, is used to define low/high risk populations and treatment decisions are made in reference to this threshold. Therefore, the sensitivity (positive predictive value) and specificity (negative predictive value) of CV risk assessment algorithms at different cut points for low and high risk should always be reported [6].

Calibration is a measure of the agreement between the predicted outcomes and actual outcomes. It is frequently assessed using either Hosmer-Lemeshow goodness of fit testing (χ^2^) or predicted to observed ratios. Goodness of fit (χ^2^) values below 20 are considered good fit, whereas predicted to observed ratios closer to 1 are considered better fit [6]. Generally, discrimination and calibration are assessed as part of internal and external validation of CV risk assessment algorithms. Table 3 outlines the results of the internal and external validation of the five non-laboratory based CV risk assessment algorithms.

##### 

**Internal validation** Internal validation is the assessment of an algorithm’s performance in the dataset from which it was derived. Although it is important in evaluating the mathematical performance and the appropriate fit of a new algorithm, it is not useful in comparing different algorithms as it is inherently biased in favor of the new algorithm [6].

The internal validation of the Framingham non-laboratory based algorithm demonstrated good discrimination (c statistic 0.749 for men and 0.785 for women) and calibration (χ^2^ 13.61 in men and 10.24 in women). Sensitivity at the highest risk threshold (>20%, 10-year risk) was approximately 48% for men and 58% for women while specificity was 85% for men and 83% for women [12].

The Gaziano non-laboratory based algorithm internal validation demonstrated good summary discrimination (C statistics 0?·?783 men and 0?·?831 women) and calibration (χ^2^ 6.61 for men and 3.45 for women). However, its sensitivity was 5.1% for women and 8.8% for men and specificity 99.5% for women and 98.6% for men at the highest risk threshold (30%, 5-year risk) [9].

The internal validation of the UK General Practice model revealed a summary C statistics of 0.67 for CVD and 0.66 for CHD. At the highest risk threshold (30%, 10-year risk) sensitivity for CVD was 38% and specificity 79% whereas for CHD sensitivity was 10% and specificity 95%. At a lower risk threshold (15%, 10-year risk) sensitivity for CVD was 85% and specificity 30%, whereas sensitivity was 44% and specificity 74% for CHD [16]. Calibration was not reported.

Internal validation of the Swedish Consultation based method revealed a good summary discrimination (C statistics 0.794) which is not gender specified [15]. However, the threshold discrimination (sensitivity and specificity) and calibration of the Consultation based method are not reported. The internal validation of the WHO/ISH non-laboratory based algorithms was not reported.

The respective internal validation outcomes indicate that the Gaziano non-laboratory based algorithm had the highest overall discrimination for men and women compared to the other algorithms. In regard to the clinically important threshold discrimination, it appears that the Framingham non-laboratory based algorithm was superior in sensitivity compared to Gaziano and the UK General Practice model. However, it is important to point out that the Gaziano non-laboratory based algorithm has the highest risk threshold compared to Framingham non-laboratory based algorithm and the UK General Practice model, and covers a shorter period of time (30%, 5-year risk). Nevertheless, even at a lower risk threshold (20%, 5-year risk) the Gaziano non-laboratory based algorithm still had low sensitivity (17.6% women, 24.8% men). At a lower risk threshold (15%, 10-year risk) the UK General Practice model sensitivity for CVD improved dramatically (from 38% to 85%) albeit at the expense of specificity.

A laboratory based algorithm [12] was derived in the same dataset as the Framingham non-laboratory based algorithm. This makes it possible to do an appropriate comparison for the internal validation. The laboratory based Framingham D’Agostino-2008 covariates include age, total cholesterol, HDL cholesterol, systolic blood pressure, antihypertensive medication use, current smoking, and diabetes status. The Framingham non-laboratory based algorithm has the same covariates but substitutes BMI for cholesterol.

When the Framingham non-laboratory based algorithm was compared to the laboratory based Framingham D’Agostino-2008, it had similar discrimination (C statistics 0.749 versus 0.763 for men and 0.785 versus 0.793 for women) and equally good calibration (χ^2^ 13.61 versus 13.48 in men and χ^2^ 10.24 versus 7.79 in women). Both algorithms were also similar in sensitivity (48% of men and 58% of women versus 49% of men and 60% of women) and specificity (85% for men and 83% for women versus 85% and 84%) respectively.

The internal validation of the Gaziano non-laboratory based algorithm also involved a comparison with a laboratory based algorithm. The Gaziano non-laboratory based algorithm was compared with the laboratory based Framingham Anderson [20] whose covariates are; age, systolic blood pressure, smoking status, total cholesterol, reported diabetes status, and current treatment for hypertension. The same risk factors are used by the Gaziano non-laboratory based algorithm but again, BMI is substituted for total cholesterol [9]. Although similar discrimination and calibration were reported between the two models (Table 3), inferences should be made with caution because the comparison was done in the dataset used to derive the Gaziano non-laboratory based algorithm but not the Framingham-Anderson algorithm. Therefore the process was inherently biased in favor of the Gaziano non-laboratory algorithm.

Likewise, the internal validation of the Swedish Consultation based method involved a comparison with the laboratory based SCORE algorithm [21], and an extensive laboratory based method [15]. The Swedish Consultation based method covariates include age, sex, present smoking, diabetes, treated hypertension, measured blood pressure, waist/height ratio and family history of CVD (angina, myocardial infarction and stroke). The SCORE covariates were; sex, age, present smoking, systolic blood pressure and total cholesterol, while the extensive laboratory based method included; age, sex, present smoking, blood pressure at baseline, waist/height ratio, family history of CVD, serum triglycerides, serum LDL/HDL-cholesterol, blood glucose, IGF-I, CRP and SDMA.

The Swedish Consultation based method was superior to SCORE in discrimination (C-statistic 0.794 vs 0.767 respectively), but the Extensive laboratory based method had the highest discrimination (C-statistic 0.806). Again, the higher discrimination observed with the Swedish Consultation based method this comparative analysis should be interpreted with caution, for it could be a result of the comparison being done in the same dataset used to derive the Swedish Consultation based method. At the same time, the clinical value of the small improvement in discrimination seen in the Extensive laboratory based method should be weighed against the costs and inconveniences of adding the extensive laboratory measures.

The internal validation of the UK General Practice model also involved a comparison with the laboratory based Framingham-Anderson [20] described earlier and an expanded Framingham that included C-reactive protein and fibrinogen. The covariates of UK General Practice model included age, systolic blood pressure, smoking habit, and self-rated health [16]. The UK General Practice model had similar discrimination with Framingham-Anderson (C statistics 0.67 versus 0.66) and the Expanded Framingham (0.64). However, the three models fell below the commendable C-statistic of 0.75 [16]. The low discrimination is a major limitation of the UK General Practice model, especially in the backdrop of the authors’ acknowledgement that the discrimination or the calibration of the model may worsen if it were used for prediction in independent data. A summary of sample characteristics, statistical methods and validation of the five non-laboratory based CV risk prediction algorithms is presented in Table 3.

##### 

**External validation** External validation is the assessment of the performance of an algorithm in an external dataset. It is considered a more appropriate approach in assessing performance and applicability of an algorithm to different populations because baseline survival and risk factors definitions used in the test are not a perfect match for those in the algorithm’s derivation dataset [6].

The Gaziano non-laboratory based algorithm was externally validated in a sample of 5,999 individuals drawn from NHANES III dataset [13]. Pandya and colleagues [13] justify use of NHANES III dataset for external validation by observing that sampling for each wave of NHANES was conducted separately, and none of the individuals from the NHANES I sample were intentionally included in the NHANES III sample. Although NHANES III was a crossectional study done between 1988 to 1994, CVD and CHD events were ascertained using cause-specific mortality which was available for adults up to 2006 [13].

The performance of the Gaziano non-laboratory based algorithm was compared with two Framingham laboratory based algorithms (Framingham-D’Agostino-2008 [12]; Framingham-Anderson [20]) and the Systematic COronary Risk Evaluation (SCORE) algorithms [21] for high and low risk settings using cause-specific mortality data. For the SCORE algorithms age was used as a measure of exposure time to risk rather than a risk factor [21]. The covariates of the algorithms have been described in the previous section.

Risk discrimination for each algorithm was assessed using the algorithm-specific ranks, with 10-year CVD death as the outcome of interest. The Gaziano non-laboratory based algorithm had a C-statistics of 0.782 in men, and 0.807 in women. In men, there was no significant difference in risk discrimination between the Gaziano algorithm and the other four laboratory-based algorithms. However in women, the Gaziano non-laboratory based algorithm had statistically significant lower C-statistics compared to SCORE and Framingham-D’Agostino-2008 [13]. Table 3 tabulates the C-statistics of the four laboratory based algorithms compared with the Gaziano non- laboratory based algorithm.

The agreement between the algorithms based on the risk threshold by the Adult Treatment Panel (APT) III guidelines (10-year Framingham CHD risk >10%) was high. Overall, 42.2% of men and 18.8% of women in the study sample would be characterized as “high” risk. In this sample, 91.9% of men and 94.6% of women were consistently ranked as “high” or “low” risk by either the Gaziano or laboratory based Framingham-D’Agostino-2008 algorithms. Similar high agreements were noted with the other three laboratory based algorithms as described in Table 3. However, the external validation of the Gaziano non-laboratory based algorithm did not assess calibration due to lack of data on non-fatal events [13].

No articles were found focusing on external validation of the Framingham non-laboratory based algorithm, WHO/ISH non-laboratory based algorithms, Swedish Consultation model or the UK General Practice model. However, two studies were found questioning the accuracy of risk classification by the WHO/ISH non-laboratory based algorithms, the major concern being an observation that the algorithms are not detailed enough for medium and high-risk patients [22,23].

#### Usability of the algorithms

The value of a risk assessment algorithm depends on the extent to which it is applicable for clinical practice. In clinical decision-making, clinicians are more likely to use risk assessment algorithms that are quick and easy to use [6,9]. The need for simple, user friendly risk algorithms is even more acute in resource constrained settings where non-physician health workers are increasingly being entrusted with traditionally physician responsibilities such as screening for and managing chronic diseases.

The Gaziano non-laboratory based algorithm is available in a gender specific chart format. All covariates of the algorithm except treatment status for blood pressure are included in the chart [9]. It is also worth noting that there is an apparent overlap, in the published charts, of the BMI categories. For instance a BMI of 25 may be placed in either category 20–25 or 25–30. This overlap can be confusing in clinical assessment even though the authors might have a plausible explanation of how to deal with the apparent overlaps.

The WHO/ISH non-laboratory based algorithms are also available in gender specific charts which are customized for use according to the six WHO regions [3]. The charts include all the covariates of the algorithm. Unlike the Gaziano and WHO/ISH non-laboratory based algorithms which are available in charts, the Framingham non-laboratory based algorithm is available in an excel spreadsheet format and an interactive online calculator [12]. All risk factors are included in the spreadsheet and electronic formats of the algorithm. The formats of the Swedish Consultation based model and the UK General Practice model are not specified.

Generally, charts are preferable to tables because they are easy to use and inexpensive to produce [6]. Additionally, the charts provide an optimal visual aid when explaining the implications of elevated risk and treatment options to the patient [10]. A randomized trial conducted in 37 medical practices in Scotland revealed that physicians and nurses preferred charts to numerical tables because they were easy to use within the time constrains of typical clinic visits [24]. Therefore, concerning the ease of clinical usability, the Gaziano and WHO/ISH charts are likely to be preferred to the spreadsheet and electronic version of Framingham non-laboratory based algorithm. Even when an electronic version of an algorithm is easy to use, its applicability in low resource settings could be difficult due to technological challenges.

For charts to attain their optimal utility Beswick and colleagues [10] contend that they ought to include all the risk factors present in the full prediction model. The Gaziano algorithm could be limited in this aspect because its charts appear to omit blood pressure treatment status which is included in the full prediction model, although this could have been factored in the derivation of the charts.

#### Inclusion of appropriate risk factors

Although net reclassification index is considered a more accurate measure for assessing the value of additional risk factors compared to C-statistic [6,25], none of the five non-laboratories based algorithm used it. All except the WHO/ISH non-laboratory based algorithms reported their C-statistic. Overall, C-statistic has been the most popular metric for assessing the value of additional risk factors [25].

In the Swedish Consultation model, the appropriateness of additional risk factors for CVD was evaluated by Petersson and colleagues [15]. When serum triglycerides, serum LDL-cholesterol/serum HDL-cholesterol, blood glucose, IGF-I, CRP and SDMA were added to the Consultation based model, only a slight improvement of the C-statistic was observed (C-statistic 0.794 vs. 0.806). May and colleagues [16] reported that the UK General Practice model was superior to an expanded Framingham function that included C-reactive protein and fibrinogen (C-statistic 0.67 vs. 0.64). As Petersson and colleagues [15] correctly observe, it is not cost-effective or clinically prudent to do such elaborate laboratory tests which are not associated with any significant improvement in risk discrimination [15].

Recognizing that all the potential risk factors for CVD cannot be included in any risk assessment algorithm, many authors have supported simplification of the risk algorithms to enhance clinical utility. However, Beswick and colleagues [10] caution against the other extreme end of the continuum, oversimplification. They contend that failure to include adequate known risk factors in an algorithm inevitably leads to unfavorable sensitivity and specificity. This is a possible predicament of the WHO/ISH non-laboratory based algorithms. Unlike the Gaziano and the Framingham non-laboratory based algorithms, which substitute BMI for cholesterol, the WHO/ISH non-laboratory based algorithms omits cholesterol measures without any substitution. Although the real implications of the simplification are unknown because no information on discrimination or reclassification has been provided for the WHO/ISH non-laboratory based algorithms, there is real potential for sensitivity and specificity to be unfavorably affected.

#### Measurable health gains

Although there is general consensus that global risk assessment is an important adjunct to CVD prevention and management, there remains considerable debate about the tangible clinical benefits associated with the use of cardiovascular risk algorithms [10]. Critics observe that small randomized trials have been heterogeneous on measurable health gains associated with use of cardiovascular risk assessment algorithms. However, most of these randomized trials have reported a small but significantly greater CVD risk reduction in the intervention groups [6].

No randomized trials were found specifically assessing the tangible health benefits associated with the use of the non-laboratory based risk assessment algorithms. However the beneficial trend observed with the laboratory based risk assessment algorithms can reasonably be expected to occur with the implementation of the non-laboratory based risk assessment algorithms. This may be especially true in low resource countries where the use of these tools could have an important role in directing preventive and treatment efforts toward those in greatest need.

### Limitations

It is possible that our search strategy using the four databases may not have included studies published elsewhere. However, the selected databases are widely used, and are well known for their comprehensive repository of high quality peer reviewed medical articles. Another potential limitation is that this systematic review may have been constrained by reliance on the published results of the reviewed algorithms. The authors of the algorithms might have carried out additional analyses relative to Cooney’s criteria, but the information was not publically available to include in this systematic review.

### Summary

The Gaziano and Framingham non-laboratory based algorithms met the majority of the specified criteria for appropriateness of statistical methods used to derive the algorithms and endpoints. Both were derived from adequately powered samples using valid statistical methods. In addition, their derivation samples were inclusive of men and women of all adult age groups, and had clearly defined endpoints. Whereas the Swedish Consultation based method and the UK General Practice model were also derived using valid statistical methods, their derivation samples were small which may have denied them adequate statistical power. In addition, the samples were not inclusive as evidenced by the UK General Practice model’s failure to include men, and younger and middle aged women; and the Swedish Consultation based method’s failure to include younger and older adult populations. Only the Gaziano non-laboratory based algorithm was derived from a racially and ethnically diverse sample. This may not be a major shortcoming for other algorithms because convincing evidence has been presented showing that CV risk prediction algorithms can be applicable to populations with different racial and ethnic profiles. No data were available to evaluate the WHO-non-laboratory based algorithms on this criterion.

When performance of the algorithms in their derivation datasets was measured based on the recommended C statistic of 0.75, the Swedish Consultation based method, Framingham and Gaziano non-laboratory based algorithm demonstrated good discrimination, but the UK General Practice model had poor discrimination. The Framingham non-laboratory based algorithm demonstrated superior sensitivity and specificity compared to the other algorithms. The performance and applicability of most of the algorithms in different populations could not be ascertained because only the Gaziano non-laboratory based algorithm was externally validated, where it demonstrated good discrimination and calibration. No data were available to evaluate the WHO-non-laboratory based algorithms on this criterion.

The criterion for usability of algorithms was adequately met by the Gaziano and WHO non-laboratory based algorithms. Both had chart formats which are considered simple and user friendly for clinical application. The Framingham non-laboratory based algorithm is only available in spreadsheet and interactive online calculator format, thus limiting its use in low technology settings. The Swedish Consultation model and UK General Practice model had no published charts or calculators which could enable their use in clinical settings.

The ability of additional CVD risk factors to improve discrimination was assessed for all algorithms except for the WHO non-laboratory based algorithms. Overall, only a slight non-cost-effective improvement of the C-statistic was observed. This indicates that the inclusion of appropriate risk factors criterion was met by all the algorithms except the WHO non-laboratory based algorithm which incorporated similar risk factors with the Gaziano non-laboratory based algorithm, but dropped cholesterol instead of substituting it with BMI or other equivalent measure.

No data were found assessing tangible health benefits associated with the use of any of the non-laboratory based risk assessment algorithms in clinical settings. However, tangible benefits can be anticipated in resource constrained primary care settings where the algorithms have an important role in directing preventive and treatment efforts toward those in greatest need.

## Conclusion

Both the Gaziano and Framingham non-laboratory based algorithms met most of the benchmarks outlined by Cooney and colleagues as hallmarks of a clinically robust risk assessment algorithm. Although the WHO/ISH non-laboratory based algorithms were designed for use in resource constrained settings, very little information has been availed to assess their performance even in their derivation dataset. The Swedish Consultation based method and the UK General Practice model are also limited by lack of charts or calculators which can be used in clinical settings to assess absolute CVD risk. External validation of the non-laboratory based risk assessment algorithms in diverse populations will be an important step in ascertaining their performance and applicability to different populations, and to enhance clinicians’ confidence in using them to guide screening and management of CVD in resource constrained settings.

## Competing interests

The authors declare that they have no competing interests.

## Authors’ contributions

JKK conceived of the study and drafted the manuscript, while EMS, SGL and LLH were involved in revising it to enhance its scientific content. All the authors have given final approval of the version to be published.

## Pre-publication history

The pre-publication history for this paper can be accessed here:

http://www.biomedcentral.com/1471-2261/13/123/prepub
